# Modeling of longitudinal polytomous outcome from complex survey data - application to investigate an association between mental distress and non-malignant respiratory diseases

**DOI:** 10.1186/1471-2288-9-84

**Published:** 2009-12-17

**Authors:** Punam Pahwa, Chandima P Karunanayake

**Affiliations:** 1 Canadian Centre for Health and Safety in Agriculture, University of Saskatchewan, 103 Hospital Drive, Saskatoon, SK, S7N OW8, Canada; 2 Department of Community Health and Epidemiology, University of Saskatchewan, 103 Hospital drive, Saskatoon, SK, S7N OW8, Canada

## Abstract

**Background:**

The data from longitudinal complex surveys based on multi-stage sampling designs contain cross-sectional dependencies among units due to clustered nature of the data and within-subject dependencies due to repeated measurements. Special statistical methods are required to analyze longitudinal complex survey data.

**Methods:**

Statistics Canada's longitudinal National Population Health Survey (NPHS) dataset from the first five cycles (1994/1995 to 2002/2003) was used to investigate the effects of demographic, social, life-style, and health-related factors on the longitudinal changes of mental distress scores among the NPHS participants who self-reported physician diagnosed respiratory diseases, specifically asthma and chronic bronchitis. The NPHS longitudinal sample includes 17,276 persons of all ages. In this report, participants 15 years and older (n = 14,713) were considered for statistical analysis. Mental distress, an ordinal outcome variable (categories: no/low, moderate, and high) was examined. Ordered logistic regression models based on the weighted generalized estimating equations approach were fitted to investigate the association between respiratory diseases and mental distress adjusting for other covariates of interest. Variance estimates of regression coefficients were computed by using bootstrap methods. The final model was used to predict the probabilities of prevalence of no/low, moderate or high mental distress scores.

**Results:**

Accounting for design effects does not vary the significance of the coefficients of the model. Participants suffering with chronic bronchitis were significantly at a higher risk (OR_adj _= 1.37; 95% CI: 1.12-1.66) of reporting high levels of mental distress compared to those who did not self-report chronic bronchitis. There was no significant association between asthma and mental distress. There was a significant interaction between sex and self-perceived general health status indicating a dose-response relationship. Among females, the risk of mental distress increases with increasing deteriorating (from excellent to very poor) self-perceived general health.

**Conclusions:**

A positive association was observed between the physician diagnosed self-reported chronic bronchitis and an increased prevalence of mental distress when adjusted for important covariates. Variance estimates of regression coefficients obtained from the sandwich estimator (i.e. not accounting for design effects) were similar to bootstrap variance estimates (i.e. accounting for design effects). Even though these two sets of variance estimates are similar, it is more appropriate to use bootstrap variance estimates.

## Background

### A methodological introduction to the statistical models

Statistics Canada has engaged in conducting large scale longitudinal surveys [1] over long periods of time. The selection of the sample of individuals who participate in such surveys is based on complex multi-stage sampling designs. Participants in these surveys have repeated measurements on the response variables of interest and several covariates over time, which lead to the dependent observations, as encountered in standard longitudinal studies [2]. The complex multi-stage sampling designs used for these longitudinal surveys also contain cross-sectional dependencies among units (caused by inherent hierarchies in the data) in addition to the within-subject dependencies due to repeated measurements. The three important characteristics of multi-stage complex surveys are stratification, clustering and unequal probability of selection, which should be accounted for at the analysis stage in order to obtain valid estimates of regression coefficients and their standard errors. As previously reported [3,4] for longitudinal dichotomous outcome, model-based analytical approach of complex survey data sets ignores stratification and clustering and may lead to biased results. In contrast, design-based analytical approach accounts for stratification and clustering by computing robust variance estimates. In this report we extended the statistical modeling of dichotomous outcome data [4] to polytomous outcome data obtained from longitudinal complex surveys. Polytomous ordinal outcomes can be treated as an extension of binary outcomes; however logistic regression models for binary outcomes extended to analyze ordinal outcomes face a number of issues specific to the ordinal case [5,6]. For a polytomous ordinal outcome, the most popular model is the logit model based on the concept of cumulative logits. Two different sets of models for ordinal outcome were utilized in this article. The first set of models (known as model-based) was based on the assumption that the study design involved only subject-level clustering due to repeated measurements and was based on the Generalized Estimating Equations (GEEs) approach [7,8], thus ignoring the complexities of the survey design. The second set of models (known as design-based) was based on the assumption that the study-design was a complex survey and incorporated complexities of the design in addition to the subject-level clustering which was an assumption for the first set of models. For the second set of models, the GEEs approach was used for the parameter coefficients estimation and complexities of the design (stratification and clustering) were incorporated via appropriate variance estimation approaches. The two commonly used approaches for producing variance estimates for estimated regression coefficients are, the analytical technique [9,10] and a replication approach [9,11]. The bootstrap method based on the replication approach was used in this article for the variance estimation.

### An application: Association between non-malignant respiratory diseases and mental health

Many etiologies have been proposed to investigate the relationship between asthma and mental health, and according to some researchers asthma develops in reaction to mental health problems, while others have proposed that depression develops in reaction to asthma [12]. It has been shown that patients diagnosed with lifetime severe asthma are more likely to have increased risk of many mental disorders, such as anxiety disorder, panic disorder, panic attacks, social phobia and specific phobia, generalized anxiety disorder and bipolar disorder [13]. A recent study based on survey conducted as a part of Centers for Disease Control and Prevention 2004 Behavioral Risk Factor Surveillance System reported that there exists an interaction among income, race/ethnicity, and asthma on mental health outcomes [14]. Chun et al. investigated the relationship between current asthma and mental health in the US population sample obtained from the 2006 Behavioral Risk Factor Surveillance System survey, and reported that there is a dose-response relationship between asthma and mental health [15].

Results based on a population-based prospective study of Dutch employees revealed that employees with the presence of chronic bronchitis were more likely to develop anxiety, and as well as depression compared to employees without these respiratory complaints [16]. Wagena et al. investigated whether or not there is an interaction among the presence of chronic bronchitis, smoking cigarettes, and psychiatric disorders [17]. Based on their data, the authors reported that smoking cigarettes is an effect modifier in the relationship between the presence of chronic bronchitis and depression and anxiety [17]. Their findings suggested that the risk for depression and anxiety increased in employees with chronic bronchitis who currently smoked or used to smoke compared to non-smokers.

Many indicators of mental health status are measured on ordinal scales. Statistics Canada's longitudinal NPHS provided a wealth of new data, which allowed us to investigate the effects of demographic variables, social, life-style, health-related and other factors on the longitudinal changes of mental distress scores among the NPHS participants who self-reported physician diagnosed respiratory diseases, specifically asthma and chronic bronchitis.

The two objectives of this manuscript are: (i) to compare the two approaches (model vs. design-based) commonly used to analyze complex survey data, and (ii) to apply these techniques to analyze real life polytomous outcome data obtained from the longitudinal complex survey in order to investigate the relationship between mental distress and non-malignant respiratory diseases.

## Methods

### Study Population

The Canadian NPHS was launched in the mid 90's [1]. The longitudinal sample consist of 17,276 participants (Newfoundland: 1082; Prince Edward Island: 1037; Nova Scotia: 1085; New Brunswick: 1125; Québec: 3000; Ontario: 4307; Manitoba: 1205; Saskatchewan: 1168; Alberta: 1544; British Columbia: 1723) at the start of Cycle 1 (1994/1995). NPHS is a sample survey with a cross-sectional design and a longitudinal follow-up. A stratified multi-stage sampling design was used to collect data from all the provinces except for Quebec. Details of this sampling design can be found elsewhere [18,19]. Briefly, each province was divided into three types of areas: Major Urban Centres, Urban Towns and Rural Areas. From each area separate geographic and/or socio-economic strata were formed. In most strata, clusters were selected with probability proportional to size (PPS). The sample of dwellings was obtained from these sample clusters. For Quebec: the province was divided geographically by crossing fifteen health areas with four urban density classes (the Montreal Census Metropolitan Area, regional capitals, small urban agglomerations, and the rural sector). In each area, clusters were defined using socio-economic characteristics and selected using a PPS sample. Selected clusters were enumerated and random samples of their dwellings were drawn: ten per cluster in major cities, twenty or thirty elsewhere.

In every participating household, one person provided demographic, socio-economic and health information about each household member for the general component of the survey. One randomly selected individual was chosen to provide in-depth information about his or her own health for the health component of the survey, and was followed for the longitudinal component of the survey [18,19]. This group of individuals will be surveyed every two years in future until 2014. For this report we used a subsample of NPHS who are 15 years and older and sample size of n = 14,713.

### Statistical models

Ordered logistic regression models [4,20] were used to predict the relationship between an ordinal mental distress outcome and a set of explanatory variables.(1)

where β_0*l *_(*l *= 1,2) are the intercepts and β_*i*_'s are regression coefficients for the covariates  and .  represents time-dependent *i*^*th *^covariate for *j*^*th *^subject at *k*^*th *^cycle (*k *= 1,2, ..., 5).  represents time-independent *i*^*th *^covariate for *j*^*th *^subject measured at the baseline. *β*_01 _is the intercept for log odds of having high distress versus moderate or no/low distress and *β*_02 _is the intercept of log odds of having high or moderate distress vs. no/low distress. The basic assumption made to conduct this type of analysis was that the regression lines for the different outcome categories were parallel to each other but were allowed to have different intercepts. This assumption was satisfied when tested by a graphical method [21].

### Estimation of regression coefficients

The SAS procedure PROC GENMOD [20] was used to fit the multivariable model in order to determine the significant predictors of mental distress. The longitudinal weight variable computed by the methodologists of Statistics Canada was used in the WEIGHT statement of SAS syntax. Currently, for ordinal outcome SAS PROC GENMOD has only one option available for specifying the within subject correlation and that is 'independent". The estimates of regression coefficients for the ordinal logistic regression model given in equation (1) above were obtained by solving the set of score equations based on multivariate quasi-likelihood approach [5,6] modified for complex survey designs using the weight variable.

### Variance estimation

#### Robust Variance estimation based on the GEEs and not accounting for the design

(model-based variance estimation). Robust variance estimation in GENMOD is based on Zeger and Liang's method [5,6] which accounts only for the within-subject dependencies due to the repeated measurements over time. The variance estimation was based on the formula given by Liang and Zeger [5,6].

#### Survey Bootstrap for Variance Estimation accounting for the design complexities

(design-based variance estimation). Statistics Canada releases design information for variance estimation only in the form of bootstrap weights: cross-sectional weights and longitudinal weights (adjusted for non-response) that have been created from taking numerous bootstrap samples of primary sampling units from the original sample. Computation of replicate survey weights is done by the methodologists of Statistics Canada who are most familiar with the survey design and the computation of weights [22]. A Bootstrap replication method was used that made appropriate use of these longitudinal bootstrap weights for the variance estimation of regression estimates. To account for the complexities of the multi-stage stratified clustered design the BOOTVAR program which was originally developed by Statistics Canada and modified by Lam (Lam M: Personal Communication) was used for the variance estimation.

### Prediction of probabilities for three different mental distress categories

Once the model was fitted, the following two predictive models were used to determine the predicted probabilities for: i) high mental distress category (p_1_); ii) moderate mental distress category (p_2_) and iii) no/low distress category (p_3_)(2)

Total probability attributable to the three distress categories is equal to 1, i.e.(4)

Equations (2) to (4) were solved to estimate probabilities p_1_, p_2_, and p_3_.

### Application to Longitudinal NPHS Data

The NPHS includes a set of questions designed to determine/investigate the mental health of NPHS participants. In this report, we used mental distress as a measure of mental health. The data on participants 15 years and older who participated from cycle 1 through cycle 5 (1994/1995 to 2002/2003) were used in this analysis. The age distribution of these study participants at the baseline [23] are given in Table 1. The variables used for the analysis are defined below and the NPHS questions used to define these variables are given in Appendix I (see additional file 1).

**Table 1:** Age distribution of participants at the baseline.

Age group (15 and older)	Frequency (%)
15-19 years	1043 (7.1)
20-24 years	1215 (8.3)
25-29 years	1337 (9.1)
30-34 years	1668 (11.3)
35-39 years	1535 (10.4)
40-44 years	1288 (8.8)
45-49 years	1223 (8.3)
50-54 years	960 (6.5)
55-59 years	869 (5.9)
60-64 years	835 (5.7)
65-69 years	820 (5.6)
70 years and over	1920 (13.0)

**Total (15 and older)**	**14713 (100.0)**

#### Dependent Variable

The mental distress variable was derived from a set of questions designed by Kessler et al. [24,25], and distress is defined as:

*'Distress*, as measured in the 1994/1995 NPHS, is a state characterized by symptoms of anxiety and depression. *Amount of distress *was assessed by a six-item symptom checklist yielding a score of 0-24 [26].

Distress, an ordinal outcome variable was examined using a six-item scale that assessed feelings of i) sadness, ii) nervousness, iii) restlessness, iv) hopelessness, v) worthlessness and vi) the feeling that everything was an effort within the previous month. The variable "distress scale" is based on the work of Kessler and Morczek [25] and was derived from the Composite International Diagnostic Interview. Scores on the distress scales ranged from 0 (no distress) to 24 (highly distressed). The distribution of the distress score based on six-item scale was highly skewed for the Canadian population, therefore it was not appropriate to use it as a continuous outcome. Based on previous research [26,27] and based on the suggestions of a geriatric psychiatrist, we categorized the outcome variable into three categories: i) no or low distress: 0-5; ii) moderate distress: 6-12; and iii) high distress: 13-24.

### Independent variables

Mental health is interplay among several factors, such as: demographic; socio-economic, social-support, health related, time of study and interactions between them. In this report the following variables were considered as independent variables:

#### Main risk factors of interest

presence or absence of asthma, presence or absence of chronic bronchitis. Participants were asked whether or not they have been diagnosed with one of the following chronic conditions (see additional file 1). These two variables were based on the question: have you ever been diagnosed by a health care provider with any of the following conditions:

(i) asthma

(ii) chronic bronchitis

These two variables are time dependent.

Demographic variables consist of age, sex, ethnicity, marital status, location of residence, and geographical area. Age was used as a time-dependent variable with four categories: 15-24 yrs, 25-54 yrs, 55-69 yrs and 70 yrs and older (reference category: 70 yrs and older). Ethnicity [28] was a time-independent dichotomous variable with two categories: white vs. non-white (non-white as a reference category). Marital status, a time-dependent variable was grouped into three categories: married/common law/partnership; separated/widowed/divorced; and single (reference category). Location of residence had two categories (a time-dependent variable) rural vs. urban. Geographical area (a time-dependent variable) was a nominal variable with five categories: Atlantic (Halifax, Newfoundland, New Brunswick, and Prince Edward Island); British Columbia; Prairies (Manitoba, Saskatoon, Alberta)); Quebec; and Ontario (reference category). Immigration status (a time-independent variable) was based on the place of birth: if place of birth was Canada then response to immigration status was 'no' and if response to place of birth was other than Canada then response to immigration status was 'yes'.

Socio-economic status variables consist of education and income. Education (a time-dependent variable) was a dichotomous variable with two categories: education received less than or equal to12 years and education received greater than 12 years. Income (a time-dependent) was divided into three categories based on the work of Wang and EI-Gebaly [29].

Social Support variables (a time-independent) consist of perceived social support (range: 0-12) and social involvement score (range: 0-8). The perceived social support score is computed using four questions that reflect whether respondents feel that they have someone they can confide in, someone they can count on, someone who can give them advice and someone who makes them feel loved. Social support score was divided into three categories: low (0-2); moderate (3-5); and high (6-12) for analysis. The social involvement score was divided into three categories: low (0-1); moderate (2-4); and high (5-8). This score was based on two questions: frequency of participation in organizations and frequency of attending religious services.

Life-style variables consist of participant's personal smoking history (a time-dependent variable) and household smoking status (a time-dependent variable). Personal smoking history was divided into three categories, non-smokers, ex-smokers and current smoker. Household smoking status was a dichotomous variable indicating presence or absence of smokers within a household.

Health related variable (time-dependent variable) consists of a self-perceived general health status, which had five categories: poor, fair, good, vary good and excellent (reference category: excellent). Four dummy variables for 'Cycle' were used to study the effect of time on mental distress.

## Results

Our study population (n = 14,713) consisted of the longitudinal sample of NPHS. At the baseline, 78.2% were classified with no/low distress, 19.4% with moderate distress and 2.4% with high distress. The pattern of distribution of participants in each of the five cycles is given in Table 2.

**Table 2:** Patterns of participation in the Canadian National Population Health Survey over 10 years (1994/1995 -- 2002/2003).

	Cycle I	Cycle II	Cycle III	Cycle IV	Cycle V	Frequency	Percent (%)
Participants with no missing values						8210	56.75

Participants with one missing values	x	x	x	x	.	2383	16.47
	x	x	x	.	x		
	x	x	.	x	x		
	x	.	x	x	x		
	.	x	x	x	x		

Participants with two missing values	x	x	x	.	.	1541	10.65
	x	x	.	x	.		
	x	x	.	.	x		
	x	.	x	.	x		
	x	.	.	x	x		
	.	x	x	x	.		
	.	x	x	.	x		
	.	x	.	x	x		
	.	.	x	x	x		
	x	.	x	x	.		

Participants with three missing values	x	x	.	.	.	1188	8.21
	x	.	.	.	x		
	.	.	.	x	x		
	x	.	x	.	.		
	.	x	x	.	.		
	.	.	x	x	.		
	x	.	.	x	.		
	.	x	.	.	x		
	.	.	x	.	x		
Participants with four missing values	.	.	.	.	x	1144	7.91
	x	.	.	.	.		
	.	x	.	.	.		
	.	.	x	.	.		
	.	.	.	x	.		

x -- indicates that mental distress was recorded at that time point.

. -- indicates that mental distress was missing at that time point.

*There are 247 observations with all missing values. The percentage is calculated with the total of 14466 instead of 14713 (based on the age group 15 years and over).

### Baseline characteristics stratified by mental distress categories

The baseline characteristics of the study population in terms of un-weighted and weighted proportions are given in additional file 2. Based on weighted proportions, higher proportions in the moderate or high level distress categories were observed for i) respondents who self-reported asthma or chronic bronchitis that have been diagnosed by a health professional; ii) younger respondents; iii) females; iv) non-white people; v) widowed/separated/divorced or single respondents; vi) immigrants; vii) respondents living in urban areas; viii) respondents from Atlantic and Quebec regions; ix) respondents in low and middle income categories; x) respondents with low education (= 12 years); xi) respondents with low social involvement score; xii) current smokers; xiii) respondents exposed to smoke within household; and xiv) respondents with 'poor' self-perceived health status.

### Unadjusted Odds ratios

The strength of relationship between mental distress and each of the independent variables based on the GEEs approach is presented as an estimate of odds ratio (OR) and 95% confidence interval (95% CI) in Table 3 and are described below.

**Table 3:** Unadjusted odds ratio (OR) and their 95% confidence interval (95% CI) based on ordinal logistics regression of the prevalence of mental distress (Modelling probability of high distress).

	OR (95% C.I.)
**Non-Malignant Respiratory Diseases**	
Asthma:	
Yes	1.43(1.24,1.66)
No	1.00
Chronic Bronchitis:	
Yes	2.13 (1.75,2.59)
No	1.00

**Demographic Information**	
Age Group:	
15-24 years	1.94(1.70,2.22)
25-54 years	1.20(1.07,1.36)
55-69 years	0.89(0.78,1.03)
70 years and over	1.00
Sex:	
Female	1.59(1.48,1.73)
Male	1.00
Ethnicity:	
White	0.85(0.75,0.98)
Non-White	1.00
Marital Status:	
Married/Common law/Partnership	0.53(0.49,0.58)
Separated/Widowed/Divorced	0.95(0.85,1.06)
Single	1.00
Location of residence:	
Rural	0.79(0.73,0.86)
Urban	1.00
Geographical area:	
Atlantic	0.93(0.83,1.04)
British Columbia	0.97(0.86,1.10)
Prairies	0.97(0.87,1.08)
Quebec	1.43(1.29,1.58)
Ontario	1.00
Immigration status:	
Yes	1.04(0.93,1.16)
No	1.00

**Socio-economic status**	
Education level:	
Less or equal to 12 years	1.35(1.25,1.45)
Greater than 12 years	1.00
Income level:	
Low	3.04(2.71,3.42)
Middle	1.45(1.32,1.59)
High	1.00
**Social Support**	
Social Involvement Score:	
Low	1.54(1.37,1.72)
Moderate	1.42(1.27,1.59)
High	1.00

**Life-style**	
Smoking Status:	
Current smoker	1.83(1.67,2.00)
Ex-Smoker	1.01(0.93,1.10)
Non-Smoker	1.00
Household Smoking:	
Yes	1.72(1.60,1.85)
No	1.00

**Health-Related**	
General Health status:	
Poor	17.21(14.20,20.86)
Fair	6.03(5.30,6.87)
Good	2.84(2.55,3.17)
Very Good	1.53(1.38,1.69)
Excellent	1.00

**Time point:**	
Cycle 5	0.68(0.62,0.74)
Cycle 4	0.63(0.59,0.68)
Cycle 3	0.72(0.67,0.78)
Cycle 2	0.70(0.66,0.75)
Cycle 1	1.00

### Non-malignant Respiratory Diseases and Mental Distress

Respondent who self-reported a physician diagnosed asthma or chronic bronchitis were more likely to have mental distress compared to those who said no to either of these chronic conditions.

### Demographic variables and mental distress

There was a dose-response relationship between age groups and mental distress with a risk of high-level distress decreasing with age [OR = 1.94; 95% CI: 1.70, 2.22 for age group 15-24 years, OR = 1.20; 95% CI: 1.07, 1.36 for age group 25-54 years, and OR = 0.89; 95% CI: 0.78, 1.03 for age group 55-69 years]. Females were at a higher risk to have high level of mental distress compared to males [OR = 1.59; 95% CI: 1.48, 1.73].

White people were significantly less likely to have a high level of distress compared to non-white people [OR = 0.85; 95% CI: 0.75, 0.98]. Compared to single respondents, married/living with common law/living with a partner [OR = 0.53; 95% CI: 0.49, 0.58] were significantly less likely to have mental distress. There is no difference between immigrant respondents compared to non-immigrant participants. Rural respondents were significantly less likely to have high level of mental distress compared to their urban counterpart. Quebec respondents were significantly at a high risk of reporting high level of distress [OR = 1.43; 95% CI: 1.29, 1.58] compared to participants from the province of Ontario.

### Socio-economic status and mental distress

Respondents with lower education (< 12 years) were significantly more likely [OR = 1.35; 95% CI: 1.25, 1.45] to report high level of distress compared to those who had high education (= 12 years). There was a dose-response relationship between income and mental distress [OR = 3.04; 95% CI: 2.71, 3.42 for low income category and OR = 1.45; 95% CI: 1.32, 1.59 for middle income category] with risk of high mental distress decreases as income increases.

### Social Support

A dose-response relationship was observed between this variable and mental distress. The strength of this relationship decreased with the increase in social involvement score [OR = 1.54; 95% CI: 1.37, 1.72 for low social involvement score and OR = 1.42; 95% CI: 1.27, 1.59 for moderate social involvement score with high social involvement score as s reference category].

### Life style variables and mental distress

Current smokers were significantly at a high risk of having high mental distress [OR = 1.83; 95% CI: 1.67, 2.00] compared to non-smokers. Those who were exposed to smoking within household were significantly more likely to report high level of mental distress [OR = 1.72; 95% CI: 1.60, 1.85] compared to those who were not exposed to smoke within their household. There was a dose-response relationship between self-perceived health status and mental distress, the risk [OR = 17.21; 95% CI: 14.2, 20.86] for those who said 'poor' to self-perceived health status decreased to [OR = 1.53; 95% CI: 1.38, 1.69] who said 'very good' to self-perceived health status.

### Multivariable Model

Additional file 3 summarizes the results from the multivariable model to assess the relationship between non-malignant respiratory diseases and mental health adjusting for important covariates: demographic, socio-economic, social-support, lifestyle, self-perceived general health status and time (cycle) and the effects of interactions using the generalized estimating equations approach. These covariates for the multivariable model were selected based on standard model building strategies [30]. The standard errors of regression coefficients were first computed by using two variance estimation methods; first based on the sandwich estimator formula given by Liang and Zegar [5], which ignores the design complexities (known as model-based methods), and second based on the bootstrap re-sampling technique, which accounts for the complexities of stratified multi-stage design (known as design based methods) were computed. The standard errors obtained by the two methods were very similar. The results based on bootstrap variance estimation were used to interpret the effect of each independent variable adjusting for other covariates as described below.

The main risk factor of interest was non-malignant respiratory diseases (asthma or chronic bronchitis). The NPHS participants who said 'yes' to physician-diagnosed asthma were not at a high risk of mental distress when adjusted for important covariates. Participants suffering with chronic bronchitis were significantly at a higher risk (OR_adj _= 1.37; 95% CI: 1.12-1.66) of reporting high levels of mental distress compared to those who did not have chronic bronchitis. Participants in the younger age groups (15-24, 25-54, and 55-69) were significantly more likely than those 70+ to report high levels of distress with odds ratio of 3.63; (95% CI: 2.03-4.36), 2.47; (95% CI: 2.15-2.84) and 1.23; (95% CI: 1.06-1.43) respectively. White people were equally likely to have high mental distress [OR_adj _= 0.97; (95% CI: 0.82-1.15)] compared to non-white people after adjusting for other socioeconomic factors.

Rural participants were less likely [OR_adj _= 0.83; (95% CI: 0.75-0.93)] to report high level of mental distress compared to urban participants. Participants from Quebec were significantly at a higher risk [OR_adj _= 1.54; (95% CI: 1.37-1.74)] to report high level of distress compared to Ontario participants. Immigrant participants were at a higher risk [OR_adj _= 1.12; (95% CI: 0.99-1.27)] of reporting high level of distress compared to non-immigrants with a borderline significance. Participants with low [OR_adj _= 1.13; (95% CI: 1.00-1.28)] or moderate [OR_adj _= 1.20; (95% CI: 1.07-1.35)] social involvement scores were significantly at a higher risk of reporting high level of distress compared to those participants who had high social involvement score. Current smokers [OR_adj _= 1.39; (95% CI: 1.23-1.57)] and ex-smokers [OR_adj _= 1.13; (95% CI: 1.02-1.24)] were significantly more likely to have high level of distress compared to the non-smokers.

Various interaction terms were tested in the multivariable model for statistical significance. The following interaction terms: education*income (p < 0.1), general health-status*sex (p < 0.05), and general health-status*household smoking (p < 0.1) were retained in the final model. The interactions education*income and general health-status*household were considered scientifically important and were kept in the model. The overall odds ratios for educational level ≤ 12 years*low income, educational level ≤ 12 years *middle income indicate that participants in these two categories were more likely to have had high distress compared to those who had high income and more than 12 years of education. In summary, low income is the strongest risk factor to predict mental distress; however education modifies the relationship between income and education (with borderline significance).

Female participants with self-perceived 'poor' health were at the highest risk (overall OR_adj _= 43.91) to have had high distress, followed by female participants with self-perceived 'fair' (overall OR_adj _= 11.85), 'good' (overall OR_adj _= 4.96) and 'very good' (overall OR_adj _= 2.36) general health status compared to the male participants with 'excellent' self-perceived general health status. Participants who were exposed to smoking within their household and had self-perceived 'poor' health status were at the highest risk (overall OR_adj _= 22.22) followed by those who were exposed to cigarette smoke at home and had 'fair' (overall OR_adj _= 7.3), 'good' (overall OR_adj _= 2.75) and 'very good' (overall OR_adj _= 1.71) health compared to males with 'excellent' general health status.

**Figure 1:**
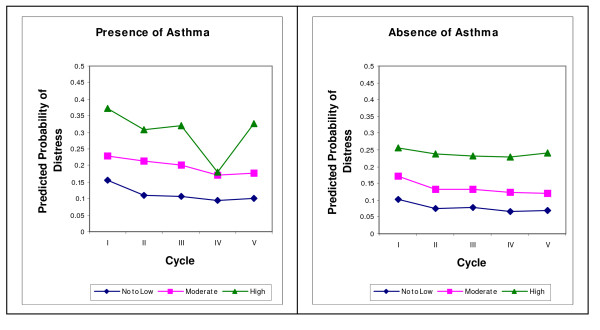
**Predicted probability of mental distress over time with presence/absence of Asthma**.

**Figure 2:**
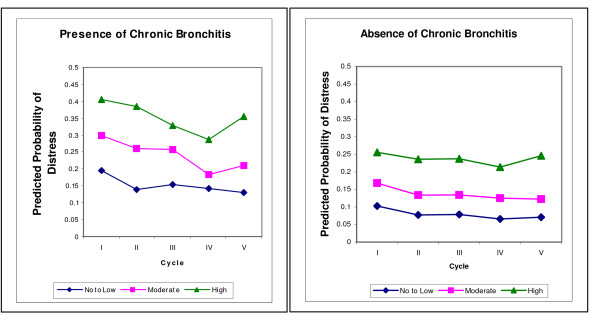
**Predicted probability of mental distress over time with presence/absence of Chronic Bronchitis**.

The predicted probability of developing no/low, moderate or high distress adjusting for other covariates is shown in Figures 1 and 2. The risk of developing any level of distress was higher in those participants who self-reported health-care professional diagnosed asthma or chronic bronchitis compared to those who did not self-report these conditions.

## Discussion

### Issues related to longitudinal complex survey data sets

There are several issues related to complex survey data analysis. First, participants in these surveys have repeated measurements of the response variables of interest and several covariates over time, which lead to the dependent observations and similar challenges of analyzing these data as encountered in standard longitudinal studies [2]. Second, the complex multi-stage sampling designs used for these longitudinal surveys also contain cross-sectional dependencies among units (caused by inherent hierarchies in the data) in addition to the within-subject dependencies due to repeated measurements, which make the statistical analyses of these data sets intricate. Third, it is very common to have missing values in longitudinal surveys. In the NPHS, several methods were used by interviewers to trace non-respondents. Non-response was mainly due to no contact or refusal by the participant. Letters were sent, second calls were made and refusals were followed up by senior interviewers to try to convince non-responders to participate. A large number of non-responders were followed up in subsequent collection periods. A detailed description can be found in Statistics Canada documentation for longitudinal surveys [1]. Missingness is an important characteristic of longitudinal studies. In this article, statistical methods used were based on the assumption that all observations were missing at random. In complex surveys it is possible to have clusters of missing data, and accounting for such clusters is a complicated and an entirely different issue, which will be attempted in another manuscript. Fourth, issues related to stratification and clustering, which are characteristics of multi-stage complex surveys. Stratification increases the variability and thus provides more precise variance estimates, while clustering decreases variability and thus variance estimates are less precise. Overall, the multi-stage design has the effect of increasing variability, thus variance estimates (if not adjusted for design complexities) are less precise compared to simple random sampling. Even though there are problems for variance estimates, the main two reasons for the popularity or acceptance of complex survey designs are that these surveys are efficient for interviewing and have better coverage of the entire region of interest [31].

Around 1970, investigators started to account for design effects in the statistical modeling via robust variance estimation procedures [32]. Coefficients of regression parameters are affected by the weights; therefore the weight variable was used to obtain consistent and valid estimates of regression parameters. An approximate method (bootstrap) was used to account for clustering and stratification, which affect variance estimates of the parameter coefficients. The approximate methods for variance estimation have been becoming popular for cross-sectional and longitudinal complex survey data analysis, and their properties have been investigated theoretically and empirically [33]. In our report, the GEE variance estimates were similar to those obtained from using the bootstrap method, which supports the following statement: '*Design-based approach reduces to the Liang-Zeger *"*Sandwich" estimator for longitudinal samples when the longitudinal units are independent' *[33]. As suggested by Binder and Roberts: *even though these two sets of variance estimates are similar, it is more appropriate to use bootstrap variance estimates because this technique accounts for design effects (stratification and clustering), bootstrap estimates were used for the purposes of formulating inferences*.

The data utilized for illustration in the present report is collected by Statistics Canada by using multi-stage complex survey design to conduct the longitudinal National Population Health survey. Data sets obtained from longitudinal complex surveys may have limitations such as initial non-response and measurement error of the covariates. Initial non-response can affect the representativeness of the sample, and measurements error of the covariates can bias the estimates significantly. For all the complex surveys (cross-sectional and longitudinal) conducted by Statistics Canada, the survey weights are computed by Statistics Canada methodologist and are provided in the data sets. These weights are computed to account for non-response, selection bias, stratification and post stratification and are used for appropriate statistical analysis [34]. There are estimation procedures which adjust for measurement error. These estimation procedures can be explored to investigate whether or not adjusting for measurement error is significantly important, which consequently can be a topic for another manuscript.

### Association between respiratory diseases and mental distress

In the present paper, we evaluated the longitudinal relationship between the presence of respiratory diseases (asthma and chronic bronchitis) and mental distress among Canadian NPHS participants who self-reported physician diagnosed asthma or chronic bronchitis, studied over a 10 year period (1994/1995 to 2001/2003). Our analysis showed that there is a positive association between the physician diagnosed chronic bronchitis and an increased prevalence of mental distress.

According to World Health Organization, depression will be the second leading contributor to of the overall burden of illness in 2020 [35]. Stephens et al. published a comprehensive report on the mental health of the Canadian population [26]. Several studies [36-40] have reported that patients with bronchial asthma have higher than expected levels of psychiatric morbidity and our results support these findings. Dales et al. [40] reported a positive association between respiratory symptoms and psychological status indicators, and these findings were supported by Janson et al. [41] with respect to respiratory symptoms. However, Janson et al. [41] were unable to observe a positive association between bronchial asthma or objective asthma-related measurements and anxiety and depression. Based on our data, those who self-reported physician diagnosed asthma were significantly at a risk of having high-level mental distress compared to those who reported no asthma. This difference was not significant when adjusted for other covariates. Similarly, NPHS participants who self-reported a physician diagnosed chronic bronchitis were significantly at a high risk of having high-level of mental distress compared to those who reported no chronic bronchitis and this difference remained significant after adjusting for the other covariates. As reported by the World Health Organization, the relationship between poverty (defined as: lack of money or material possessions) and mental health is complicated [35]. Our data showed that subjects with low income and low education were at higher risk compared to those who had high education and high income.

It is hard to explain the decreasing trend over time (Figures 1 and 2) in the predicted probabilities of developing distress for those who self-reported health-care professional diagnosed asthma or chronic bronchitis. In contrast, predicted probability of developing distress is consistent over time for those participants who did not self-report these conditions. One of the possible reasons which could lead to the decreasing trend of distress among those who self-reported health-care professional asthma or chronic bronchitis is: Our study population is a closed cohort and participants who were sick either withdrew from the survey or were moved to institutions (and hence not included in the survey) or died. In order to explore this issue we need to compare stay-in participants with those who dropped out and use some of the handling missing data approaches to conduct an appropriate statistical analysis, which is a topic of another manuscript. This needs further investigation of comparison between participants who stayed-in to those who dropped out from the survey.

## Conclusions

A positive association was observed between the physician diagnosed self-reported chronic bronchitis and an increased prevalence of mental distress when adjusted for important covariates. Variance estimates of regression coefficients obtained from the sandwich estimator (i.e. not accounting for design effects) were similar to bootstrap variance estimates (i.e. accounting for design effects). Even though these two sets of variance estimates are similar, it is more appropriate to use bootstrap variance estimates.

## Abbreviations

NPHS: National Population Health Survey; OR_adj_: adjusted Odds Ratio; GEE: Generalized Estimating Equations.

## Competing interests

The authors declare that they have no competing interests.

## Authors' contributions

It was PP's idea to compare the model-based and design-based methods by modeling polytomous outcome from longitudinal national complex survey data, and she prepared the manuscript. CPK conducted the statistical analysis. All authors read and approved the final manuscript.

## Pre-publication history

The pre-publication history for this paper can be accessed here:

http://www.biomedcentral.com/1471-2288/9/84/prepub

## Supplementary Material

Additional file 1 **Appendix I**. Questions Used to Create Variable for Statistical Analysis. Click here for file

Additional file 2 **Table S2: Baseline (Cycle I) characteristics of the National Population Health Survey Stratified by Mental distress**. This is a table of baseline characteristics of NPHS participants. Click here for file

Additional file 3**Table S3. Regression estimates (); GEE-based standard errors [*s.e*.()]_*Robust *_] and bootstrapped standard errors [*s.e*. ()_*Bootstrap*_] and adjusted odds ratio (OR_adj_) and their 95% confidence interval (95% CI) based on ordinal logistics regression of the prevalence of mental distress (Modeling probability of high distress)**. This is a table of ordinal logistics regression model results.Click here for file

## References

[B1] Statistics Canada National population health survey (NPHS), cycle 1 to 7 (1994/1995 to 2006/2007) Longitudinal Documentation [Internet] 2008 Ottawa: Statistics Canada http://www.statcan.gc.ca/imdb-bmdi/document/3225_D5_T1_V4-eng.pdf [cited 2008 Dec 20]

[B2] Diggle P Lang K Zeger S Analysis of Longitudinal Data 1995 Oxford, UK: Oxford Science Publication

[B3] Ghosh S Pahwa P Rennie DC Comparison of Design-based and Model-based methods to estimate the variance using National Population Health Survey Data (1994-2003) Model Assisted Statistics andApplications 2008 3 1 33 42

[B4] Ghosh S Pahwa P Rennie D Comparison between the design-based and model-based approaches using longitudinal survey data Model Assisted Statistics and Applications 2008 3 2 177 185

[B5] Liang K-Y Zeger Sl Longitudinal data analysis using generalized linear models Biometrika 1986 73 13 22 10.1093/biomet/73.1.13

[B6] Zeger Sl Liang K-Y Longitudinal data analysis for discrete and continuous outcomes Biometrics 1986 42 121 130 10.2307/2531248 3719049

[B7] Binder DA Roberts GA Statistical inference in survey data analysis: where does the sample design fit in? http://socserv.socsci.mcmaster.ca/rdc2003/binderoberts.pdf

[B8] Demnati A Rao JNK Linearization Variance Estimators for Survey Data Survey Methodology 2004 30 138 143

[B9] Rust K Rao JNK Variance Estimation for Complex Estimators in Sample Surveys Statistics in Medical Research 1996 5 381 397 10.1177/096228029600500305 8931197

[B10] Molenberghs G Verbeke G Models for Discrete Longitudinal Data 2005 New York, USA: Springer Science and Business Media Inc

[B11] Liu I Agresti A The analysis of ordered categorical data: An overview and a survey of recent developments Socieddad de Estadistica e Investigacion Operativa Test 2005 14 1 1 73

[B12] Goldney RD Ruffin R Fisher LJ Asthma symptoms associated with depression and lower quality of life: a population survey Med J Aust 2003 178 437 441 12720509 10.5694/j.1326-5377.2003.tb05408.x

[B13] Goodwin R Jacobi F Thefeld W Mental Disorders and Asthma in the Community Arch Gen Psychiatry 2003 60 11 1125 1130 10.1001/archpsyc.60.11.1125 14609888

[B14] Bandiera FC Pereira DB Arif AA Dodge B Asal N Race/Ethnicity, Income, Chronic Asthma, and Mental Health: A Cross-Sectional Study Using the Behavioral Risk Factor Surveillance System Psychosom Med 2008 70 1 77 84 10.1097/PSY.0b013e31815ff3ad 18158369

[B15] Chun TH Weitzen SH Fritz GK The Asthma/Mental Health Nexus in a Population-Based Sample of the United States Chest 2008 134 6 1176 1182 10.1378/chest.08-1528 18719055

[B16] Wagena EJ Van Amelsvoort LPM Kant I Wouters EFM Chronic Bronchitis, Cigarette Smoking, and the Subsequent Onset of Depression and Anxiety: Results From a Prospective Population-Based Cohort Study Psychosomatic Med 2005 67 656 660 10.1097/01.psy.0000171197.29484.6b 16046384

[B17] Wagena EJ Kant I Van Amelsvoort LPM Wouters EFM Van Schayck CP Swaen GMH Risk of Depression and Anxiety in Employees With Chronic Bronchitis: The Modifying Effect of Cigarette Smoking Psychometic Med 2004 66 5 729 734 10.1097/01.psy.0000138127.00991.cf 15385698

[B18] Kish L Mutlipurpose Sample Design. Survey Methdology 1988 14 19 32

[B19] Tambay J-L Catlin G Sample design of the National Population Health Survey Health Reports 1995 7 29 38 7578995

[B20] Stokes EM Davis CS Koch GG Categorical Data Analysis Using The SAS System Cary 2000 NC: SAS Institute

[B21] Kim Ji-H Assessing Practical Significance of the Proportional Odds Assumption http://stat.soongsil.ac.kr/~jhkim/Publication/stat&prob_2003.pdf

[B22] Yeo D Mantel H Liu T-P Bootstrap variance estimation for the National Population Health Survey http://www.amstat.org/Sections/Srms/Proceedings/papers/1999_136.pdf

[B23] Statistics Canada National population health survey (NPHS), cycle 1 to 7 (1994/1995 to 2006/2007) Data dictionary, Master File: Longitudinal square (rounded [Internet] 2008 Ottawa: Statistics Canada http://www.statcan.gc.ca/imdb-bmdi/document/3225_D11_T9_V3-eng.pdf [cited 2008 Dec 20]

[B24] National Mental Health Information Center http://www.mentalhealth.org/publications/allpubs/SMA04-3938/Chapter12.asp

[B25] Kessler RC Andrews G Colpe LJ Hiripi E Mroczek DK Zaslavsky A Short screening scales to monitor population prevalences and trends in non-specific psychological distress Psychological Med 2002 32 6 959 976 10.1017/S0033291702006074 12214795

[B26] Stephens T Dulberg C Joubert N Mental health of the Canadian population: a comprehensive analysis Chronic Dis Can 2000 20 3 118 26 10557202

[B27] Baggaley RF Ganaba R Fillippi V Kere M Marshall T Sombie I Storeng KT Patel V Detecting depression after pregnancy: the validity of the K10 and K6 in Burkina Faso Trop Med Int Health 2007 12 10 1225 9 17956505 10.1111/j.1365-3156.2007.01906.x

[B28] Ng E Altman B Berthelot J-M Racial differences in HUI-based disability using the 2003 Joint Canada/United States Survey of Health: a cross-national comparison http://paa2006.princeton.edu/download.aspx?submissionId=60679

[B29] Wang J Nady EI-Guebaly Socio-demographic factors associated with co-morbid major depressive episodes and alcohol dependence in the general population Canadian J Psychiatry 2004 49 1 37 44 10.1177/070674370404900106 14763676

[B30] Hosmer DW Lemshow S Applied Logistic Regression 1989 New York: Wiley

[B31] Beland Y MacNabb L Population Health Surveys Bootstrap Hands-on Workshop http://data.library.ubc.ca/rdc/other/0702Hands_on.ppt

[B32] Rao JNK Interplay between sample survey theory and practice: An appraisal Survey Methodology 2005 31 2 117 138

[B33] Binder DA Roberts GR Statistical Inference in survey data analysis: where does the sample design fit in? http://socserv.socsci.mcmaster.ca/rdc2003/binderoberts.pdf

[B34] Pantoja-Galicia N Thompsom ME Kovacevic M Assessing the temporal association of events using longitudinal complex surveys Technical report, University of Waterloo http://www.iser.essex.ac.uk/files/survey/ulsc/methodological-research/mols-2006/scientific-social-programme/papers/Pantoja-Galicia.pdf

[B35] World Health Organization The World Health Report 2001 Mental Health: New Understanding, New Hope 2001 Geneva: WHO

[B36] Government of Canada The Human Face of Mental Health and Mental Illness in Canada. 2006. ^© ^Minister of Public Works and Government Services Canada Cat. No. HP5-19/2006E 2006 41

[B37] Lyketsos CG Lyketsos GC Richardson SC Beis A Dsythymic states and depression syndrome in physical conditions of presumably psychogenic origin Acta Psychiatr Scand 1987 76 529 534 10.1111/j.1600-0447.1987.tb02914.x 3434324

[B38] Yellowless PM Haynes S Potts N Ruffin RE Psychiatric morbidity in patients with life-threatening asthma: Initial report of a controlled study Med J Aust 1983 67 361 370 10.5694/j.1326-5377.1988.tb120596.x 3412214

[B39] Oswald NC Waller RE Drinkwater J Relationship between breathless and anxiety in asthma and bronchitis: a comparative study British Med J 1970 2 14 17 10.1136/bmj.2.5700.14 PMC1699746 5440566

[B40] Dales RE Spitzer WO Schecter MT Suissa S The influence of psychological status on respiratory symptoms reporting AmRev Respir Dis 1989 139 1459 1463 10.1164/ajrccm/139.6.1459 2729753

[B41] Janson C Bjornsson E Hetta J Anxiety and depression in relation to respiratory symptoms and asthma Am J RespirCrit Care Med 1994 149 930 934 10.1164/ajrccm.149.4.8143058 8143058

