# Supplementary material for: Modeling of longitudinal polytomous outcome from complex survey data - application to investigate an association between mental distress and non-malignant respiratory diseases
Source: BMC Med Res Methodol. 2009 Dec 17;9:84. doi: 10.1186/1471-2288-9-84 (PMC2806372; doi:10.1186/1471-2288-9-84)
Supplement: Additional file 1 — Appendix I. Questions Used to Create Variable for Statistical Analysis. [file 1471-2288-9-84-S1.PDF]

Appendix I
Questions Used to Create Variable for Statistical Analysis
(Source: Statistics Canada documentation for the derived variables)

Outcome variable: Mental distress is based on the following questions:

MHLTH-INTa Now some questions about mental and emotional well-being. During the past month4 , about how often did you feel:

MHLTH-Q1a ... so sad that nothing could cheer you up?
(Read list. Mark one only.)
___ All of the time
___ Most of the time
___ Some of the time
___ A little of the time
___ None of the time
DK, R (Go to MHLTH-Q1k)

MHLTH-Q1b ... nervous?
(Read list. Mark one only.)
___ All of the time
___ Most of the time
___ Some of the time
___ A little of the time
___ None of the time
DK, R (Go to MHLTH-Q1k)

MHLTH-Q1c ... restless or fidgety?
(Read list. Mark one only.)
___ All of the time
___ Most of the time
___ Some of the time
___ A little of the time
___ None of the time
DK, R (Go to MHLTH-Q1k)

MHLTH-Q1d ... hopeless?
(Read list. Mark one only.)
___ All of the time
___ Most of the time
___ Some of the time
___ A little of the time
___ None of the time
DK, R (Go to MHLTH-Q1k)

MHLTH-Q1e ... worthless?
(Read list. Mark one only.)
___ All of the time
___ Most of the time
___ Some of the time
___ A little of the time
___ None of the time
DK, R (Go to MHLTH-Q1k)

MHLTH-Q1f During the past month, about how often did you feel that everything was an effort?
(Read list. Mark one only.)
___ All of the time
___ Most of the time
___ Some of the time
___ A little of the time
___ None of the time
DK, R (Go to MHLTH-Q1k)

The psychological distress scale known as the K6 scale was used. '*The K6 scale measures non-specific psychological distress and it was first used in the 1997 redesigned US National Health Interview Survey (NHIS), a household survey of adults aged 18 years and older. The items and scoring used to derive the distress score are based on the work of Kessler and Mroczek (1994, the University of Michigan.) The scale questions were developed from a pool of 612 questions drawn from existing distress and depression screening scales and refined through ratings of an expert panel, comparison of correspondence with 15 domains represented in the DSM-III-R diagnoses of major depression and generalized anxiety disorder plus the positive affect domain, and analyses of two pilot studies. The full account of the development of the item scales is found in Kessler RC, et al., 2002; http://www.hcp.med.harvard.edu/ncs/k6_scales.php'*

Independent Variables:

"long-term conditions" refer to conditions that have lasted or are expected to last 6 months or more. **CHRON-Q1** Do(es) ... have any of the following long-term conditions that have been diagnosed by a health professional:
(Read list. Mark all that apply.)

- List of chronic conditions was provided to the participant. In that list asthma and chronic bronchitis were two options.

DEMO_Q5 Enter or ask ... 's sex.

___ Male
___ Female

DEMO_Q6 What is ... current marital status?
(Note: if age < 15, marital status is automatically = single)

___ Now married
___ Common-law
___ Living with a partner
___ Single (never married)
___ Widowed
___ Separated
___ Divorced

Immigration Status is a derived dichotomous variable, which is based on the place of birth. If place of birth is Canada, then immigration status is 'No' and if place of birth is other than Canada then immigration status is 'Yes'.

Education level is derived variable with two categories: first category 'Less or equal to 12 years' and second category 'Greater than 12 years'

Income: Statistics Canada computed derived variable for the total household income in to four categories as follows:

Code	Description	Income	Household Size
1	Lowest income	Less than $15,000	1 or 2 persons
		Less than $20,000	3 or 4 persons
		Less than $30,000	5 or more persons
2	Lower middle income	$15,000 to $29,999	1 or 2 persons
		$20,000 to $39,999	3 or 4 persons
		$30,000 to $59,999	5 or more persons
3	Upper middle income	$30,000 to $59,999	1 or 2 persons
		$40,000 to $79,999	3 or 4 persons
		$60,000 to $79,999	5 or more persons
4	Highest income	$60,000 or more	1 or 2 persons
		$80,000 or more	3 persons or more
9	Not stated	Unknown income	Otherwise

We pooled lower and upper middle income categories into 'middle' income category. Hence we used three categories: Lower, Middle, and Upper.

Social Support:

There are two variables which measure social support. These derived variables are self-perceived social support and social involvement score.

Perceived Social Support

'The perceived social support index is composed of four items that reflect whether respondents feel that they have someone they can confide in, someone they can count on, someone who can give them advice and someone who makes them feel loved'. These questions were asked in the first two cycles of NPHS. Therefore we decided to use the perceived social support score computed based on these questions only for Cycle I and treated this variable as a time-independent variable. The computed variable social support score has three categories: Low, moderate, and High.

Social Involvement Score:

'The social involvement dimension is measured by two items that reflect the frequency of participation in associations or voluntary organizations and the frequency of attendance at religious services in the last year'. These questions were asked in the first two cycles of NPHS. Therefore we decided to use the perceived social support score computed based on these questions only for Cycle I and treated this variable as a time-independent variable. The computed variable social involvement score has three categories: Low, moderate, and High.

Smoking Status: Smoking status was divided into three categories: Current Smoker, Ex-Smoker, and Non-Smoker.

Code	Description of derived variable by NPHS	Categories used in the present analysis
1	Daily smoker	Current Smoker
2	Occasional smoker but former daily smoker	Ex-Smoker
3	Always an occasional smoker	Current Smoker
4	Former daily smoker	Ex-Smoker
5	Former occasional smoker	Ex-Smoker
6	Never smoked	Non-Smoker
96	Not applicable	
99	Not stated	

Household Smoking: Dichotomous variable based on the following question:

Does anyone in this household smoke regularly inside the house?

Yes____
No____

Self-perceived general health: Based on the following question:

In general would you say your health is:

Excellent___
Very good___
Good____
Fair____
Poor____
