# Supplementary material for: Modeling of longitudinal polytomous outcome from complex survey data - application to investigate an association between mental distress and non-malignant respiratory diseases
Source: BMC Med Res Methodol. 2009 Dec 17;9:84. doi: 10.1186/1471-2288-9-84 (PMC2806372; doi:10.1186/1471-2288-9-84)
Supplement: Additional file 2 — Table S2: Baseline (Cycle I) characteristics of the National Population Health Survey Stratified by Mental distress. This is a table of baseline characteristics of NPHS participants. [file 1471-2288-9-84-S2.DOC]

**Table S2:** Baseline (Cycle I) characteristics of the National Population Health Survey Stratified by Mental distress

|  | Distress Level | | | | | |
| --- | --- | --- | --- | --- | --- | --- |
|  | No to Low | | Moderate | | High | |
|  | Unweighted  n (%) | Weighted  (%) | Unweighted  n (%) | Weighted  (%) | Unweighted  n (%) | Weighted  (%) |
| **Non-malignant respiratory diseases** |  |  |  |  |  |  |
| Asthma  Yes  No  * 1184 missing values | 545(67.2%)  10102(79.4%) | 68.6%  79.0% | 219(27.00%)  2295(18.05%) | 26.11%  18.65% | 47(5.80%)  321(2.52%) | 5.28%  2.31% |
| Chronic Bronchitis  Yes  No  * 1184 missing values | 314(62.7%)  10333(79.3%) | 59.8%  79.1% | 148(29.5%)  2366(18.2%) | 32.25%  18.64% | 39(7.8%)  329(2.53%) | 7.96%  2.30% |
| **Demographic Information** |  |  |  |  |  |  |
| Age (mean) in years | 45.77 | 43.52 | 40.13 | 38.22 | 42.39 | 40.81 |
| Age groups  15-24 years  25-54 years  55-69 years  70 years and over  * 1170 missing values | 1394(68.4%)  5835(78.6%)  1949(84.1%)  1481(83.9%) | 67.29%  79.33%  83.94%  83.33% | 574(28.18%)  1377(18.55%)  317(13.68%)  247(13.99%) | 29.50%  18.27%  14.02%  14.24% | 69(3.39%)  210(2.83%)  52(2.24%)  38(2.15%) | 3.31%  2.40%  2.04%  2.43% |
| Sex  Male  Female  * 1170 missing values | 4897(82.2%)  5762(76.0%) | 82.04%  75.18% | 938(15.74%)  1577(20.79%) | 16.16%  21.71% | 124(2.08%)  245(3.23%) | 1.81%  3.11% |
| Ethnicity  White  Non-White  * 1175 missing values | 10052(79.1%)  602(72.4%) | 78.91%  73.64% | F@  F@ | 18.55%  24.22% | F@  F@ | 2.53%  2.14% |
| Immigration Status  Yes  No  * 1178 missing values | 1443(77.66%)  9209(78.86%) | 76.78%  78.78% | 367(19.75%)  2147(18.39%) | 20.83%  18.70% | 48(2.58%)  321(2.75%) | 2.39%  2.52% |
| Marital Status  Married, Living common law, partnership  Widowed, Separated, divorced  Single  * 1172 missing values | 6202(83.27%)  2064(75.72%)  2391(71.01%) | 82.75%  74.33%  69.74% | 1110(14.90%)  545(19.99%)  860(25.54%) | 15.40%  21.56%  27.02% | 136(1.83%)  117(4.29%)  116(3.45%) | 1.85%  4.11%  3.24% |
| **Location of residence**  Rural  Urban  * 1170 missing values | 2700(81.03%)  7959(77.95%) | 82.13%  77.67% | 552(16.57%)  1963(19.22%) | 15.84%  19.74% | 80(2.40%)  289(2.83%) | 2.03%  2.59% |
| **Geographic area**  Atlantic  British Columbia  Prairies  Quebec  Ontario  * 1170 missing values | 2725(80.43%)  1096(80.29%)  2461(80.58%)  1706(72.17%)  2671(79.21%) | 80.27%  80.47%  81.01%  73.26%  79.73% | 572(16.88%)  234(17.14%)  523(17.13%)  578(24.45%)  608(18.03%) | 16.69%  17.53%  16.75%  23.76%  17.95% | 91(2.69%)  35(2.56%)  70(2.29%)  80(3.38%)  93(2.76%) | 3.05%  2.00%  2.24%  2.98%  2.32% |
| **Socio-economic status** |  |  |  |  |  |  |
| Income  Low  Middle  High  * 1716 missing values | 2116(69.51%)  6786(80.63%)  1308(85.10%) | 69.59%  79.36%  83.61% | 754(24.77%)  F@  F@ | 24.80%  18.64%  15.14% | 174(5.72%)  F@  F@ | 5.61%  2.00%  1.25% |
| Education Level  Less than 12 years  Greater or equal 12 years  * 1187 missing values | 4846(77.08%)  5800(80.12%) | 75.83%  80.50% | 1242(19.76%)  1271(17.56%) | 21.22%  17.39% | 199(3.17%)  168(2.32%) | 2.95%  2.10% |
| **Social Support** |  |  |  |  |  |  |
| Social support index  High  Moderate  Low  * 7408 missing values | 1183(84.62%)  3672(83.08%)  1075(72.29%) | 84.64%  83.11%  72.53% | F@  F@  373(25.082%) | 14.11%  15.41%  25.58% | F@  F@  39(2.62%) | 1.25%  1.48%  1.89% |
| Social Involvement Score  Low  Moderate  High  * 1190 missing values | 4082(75.49%)  4072(78.84%)  2492(84.45%) | 76.20%  77.97%  83.80% | 1148(21.33%)  942(18.24%)  418(14.16%) | 20.85%  19.34%  14.94% | 177(3.27%)  151(2.92%)  41(1.39%) | 2.94%  2.69%  1.26% |
| **Life-Style** |  |  |  |  |  |  |
| Smoking Status  Current smoker  Ex-Smoker  Non-Smoker  * 1173 missing values | 3070(71.20%)  3494(82.33%)  4092(82.10%) | 71.18%  82.01%  81.26% | 1048(24.30%)  662(15.60%)  805(16.15%) | 24.56%  16.17%  17.10% | 194(4.50%)  88(2.07%)  87(1.75%) | 4.26%  1.82%  1.64% |
| Household Smoking  Yes  No  * 1173 missing values | 3667(72.87%)  6989(82.15%) | 72.70%  81.72% | 1152(22.89%)  1363(16.02%) | 23.19%  16.72% | 213(4.23%)  156(1.83%) | 4.11%  1.56% |
| **Health-related:** |  |  |  |  |  |  |
| General Health Status  Poor  Fair  Good  Very Good  Excellent  * 1170 missing values | 150(40.21%)  826(61.37%)  2724(74.73%)  4217(83.16%)  2742(88.22%) | 36.95%  60.07%  72.78%  82.27%  88.48% | 148(39.68%)  416(30.91%)  807(22.14%)  F@  F@ | 43.52%  32.05%  24.17%  16.54%  10.95% | 75(20.11%)  104(7.73%)  114(3.13%)  F@  F@ | 19.53%  7.88%  3.05%  1.19%  0.56% |
| @ F – not reported due to confidentiality |  |  |  |  |  |  |
