# Supplementary material for: Modeling of longitudinal polytomous outcome from complex survey data - application to investigate an association between mental distress and non-malignant respiratory diseases
Source: BMC Med Res Methodol. 2009 Dec 17;9:84. doi: 10.1186/1471-2288-9-84 (PMC2806372; doi:10.1186/1471-2288-9-84)
Supplement: Additional file 3 — Table S3. Regression estimates (); GEE-based standard errors [s.e.()]Robust ] and bootstrapped standard errors [s.e. ()Bootstrap] and adjusted odds ratio (ORadj) and their 95% confidence interval (95% CI) based on ordinal logistics regression of the prevalence of mental distress (Modeling probability of high distress). This is a table of ordinal logistics regression model results. [file 1471-2288-9-84-S3.DOC]

**Table S3**. Regression estimates ($\hat{\beta}$); GEE-based standard errors [*s.e*.($\hat{\beta}$)]*_Robust_* ] and bootstrapped standard errors [*s.e*. ($\hat{\beta}$)*_Bootstra_*_p_] and adjusted odds ratio (OR_adj_) and their 95% confidence interval (95% CI) based on ordinal logistics regression of the prevalence of mental distress (Modelling probability of high distress)

|  | | $\hat{\beta}$[*s.e*($\hat{\beta}$)]*_Robust_* | $\hat{\beta}$ [*s.e* ($\hat{\beta}$)]*_Bootstra_*_p_ | | OR_adj_ (95% CI)*_GEE_* | | OR_adj_ (95% CI)*_Bootstrap_* |
| --- | --- | --- | --- | --- | --- | --- | --- |
| Intercept1 | | -6.07 [0.18] | -6.07 [0.19] | | 0.0023(0.0016,0.0033) | | 0.0023(0.0016,0.0034) |
| Intercept2 | | -3.52 [0.18] | -3.52 [0.19] | | 0.03(0.02,0.04) | | 0.03(0.02,0.04) |
| **Non-Malignant Respiratory Diseases** | |  |  | |  | |  |
| Asthma | |  | | |  | |  |
| Yes | | -0.06 [0.08] | -0.06 [0.08] | | 0.94(0.81,1.09) | | 0.94(0.80,1.10) |
| No | |  |  | | 1.00 | | 1.00 |
| Chronic Bronchitis | |  | | |  | |  |
| Yes | | 0.31 [0.10] | 0.31 [0.10] | | 1.37 (1.12,1.67) | | 1.37 (1.12,1.66) |
| No | |  |  | | 1.00 | | 1.00 |
| **Demographic Information** | |  |  | |  | |  |
| Age Group | |  |  | |  | |  |
| 15-24 years | | 1.29[0.09] | 1.29[0.09] | | 3.63(3.03,4.36) | | 3.63(3.06,4.30) |
| 25-54 years | | 0.90[0.07] | 0.90[0.07] | | 2.47(2.15,2.84) | | 2.47(2.15,2.85) |
| 55-69 years | | 0.21 [0.08] | 0.21 [0.08] | | 1.23(1.06,1.43) | | 1.23(1.05,1.43) |
| 70 years and over | |  |  | | 1.00 | | 1.00 |
| Sex | |  |  | |  | |  |
| Female | | 0.31 [0.11] | 0.31 [0.10] | | 1.36(1.10,1.68) | | 1.36(1.11,1.66) |
| Male | |  |  | | 1.00 | | 1.00 |
| Ethnicity | |  | | |  | |  |
| White | | -0.03 [0.08] | -0.03 [0.09] | | 0.97(0.82,1.15) | | 0.97(0.81,1.16) |
| Non-White | |  |  | | 1.00 | | 1.00 |
| Marital Status | |  | | |  | |  |
| Married/Common law/ Partnership | | -0.37 [0.06] | -0.37 [0.06] | | 0.69(0.62,0.78) | | 0.69(0.61,0.78) |
| Separated/ Widowed/ Divorced | | -0.01 [0.07] | -0.01 [0.07] | | 0.98(0.85,1.13) | | 0.98(0.86,1.13) |
| Single | |  |  | | 1.00 | | 1.00 |
| Location of residence | |  | | |  | |  |
| Rural | | -0.19 [0.05] | -0.19 [0.06] | | 0.83(0.75,0.91) | | 0.83(0.74,0.93) |
| Urban | |  |  | | 1.00 | | 1.00 |
| **Table 5(Contd./-)** | |  | | |  | |  |
| Geographical area | |  | | |  | |  |
| Atlantic | | -0.08 [0.06] | -0.08 [0.07] | | 0.92(0.81,1.05) | | 0.92(0.81,1.06) |
| British Columbia | | -0.01 [0.07] | -0.01 [0.07] | | 0.99(0.86,1.13) | | 0.99(0.85,1.14) |
| Prairies | | -0.04 [0.06] | -0.04 [0.06] | | 0.96(0.85,1.08) | | 0.96(0.86,1.08) |
| Quebec | | 0.43 [0.06] | 0.43 [0.06] | | 1.54(1.37,1.73) | | 1.54(1.37,1.74) |
| Ontario | |  |  | | 1.00 | | 1.00 |
| Immigration status | |  | | |  | |  |
| Yes | | 0.12 [0.06] | 0.12 [0.06] | | 1.12(0.99,1.28) | | 1.12(0.99,1.27) |
| No | |  |  | | 1.00 | | 1.00 |
| **Socio-economic status** | |  | | |  | |  |
| Education level | |  | | |  | |  |
| Less or equal to 12 years | | 0.15 [0.11] | 0.15 [0.12] | | 1.17(0.94,1.45) | | 1.17(0.92,1.48) |
| Greater than 12 years | |  |  | | 1.00 | | 1.00 |
| Income level | |  | | |  | |  |
| Low | | 0.56 [0.09] | 0.56 [0.09] | | 1.74(1.46,2.08) | | 1.74(1.46,2.08) |
| Middle | | 0.11 [0.07] | 0.11 [0.07] | | 1.11(0.98,1.27) | | 1.11(0.97,1.28) |
| High | |  |  | | 1.00 | | 1.00 |
| **Social Support** | |  | | |  | |  |
| Social Involvement Score | |  |  | |  | |  |
| Low | | 0.12 [0.06] | 0.12 [0.06] | | 1.13(1.00,1.28) | | 1.13(1.00,1.28) |
| Moderate | | 0.18 [0.06] | 0.18 [0.06] | | 1.20(1.06,1.35) | | 1.20(1.07,1.35) |
| High | |  |  | | 1.00 | | 1.00 |
| **Life-style** | |  | | |  | |  |
| Smoking Status | |  | | |  | |  |
| Current smoker | | 0.33 [0.07] | 0.33 [0.06] | | 1.39(1.22,1.58) | | 1.39(1.23,1.57) |
| Ex-Smoker | | 0.12 [0.05] | 0.12 [0.05] | | 1.13(1.02,1.24) | | 1.13(1.02,1.24) |
| Non-Smoker | |  |  | | 1.00 | | 1.00 |
| Household Smoking | |  | | |  | |  |
| Yes | | 0.22 [0.10] | 0.22 [0.11] | | 1.25(1.01,1.53) | | 1.25(1.01,1.54) |
| No | |  |  | | 1.00 | | 1.00 |
| **Table 5 (Contd./-)** | |  | | |  | |  |
| **Health- Related:** | |  | | |  | |  |
| General Health status | |  | | |  | |  |
| Poor | | 2.90 [0.18] | 2.90 [0.18] | | 18.14(12.85,25.62) | | 18.14(12.71,25.89) |
| Fair | | 1.90 [0.14] | 1.90 [0.13] | | 6.70(5.13,8.75) | | 6.70(5.20,8.62) |
| Good | | 1.14 [0.11] | 1.14 [0.10] | | 3.12(2.51,3.89) | | 3.12(2.55,3.82) |
| Very Good | | 0.50 [0.11] | 0.50 [0.11] | | 1.65(1.33,2.03) | | 1.65(1.34,2.03) |
| Excellent | |  | | | 1.00 | | 1.00 |
| **Time point** | |  | | |  | |  |
| Cycle 5 | | -0.40 [0.05] | -0.40 [0.05] | | 0.67(0.60,0.74) | | 0.67(0.60,0.74) |
| Cycle 4 | | -0.53 [0.05] | -0.53 [0.05] | | 0.59(0.53,0.65) | | 0.59(0.53,0.65) |
| Cycle 3 | | -0.28 [0.04] | -0.28 [0.05] | | 0.76(0.69,0.83) | | 0.76(0.68,0.84) |
| Cycle 2 | | -0.36 [0.04] | -0.36 [0.04] | | 0.69(0.64,0.75) | | 0.69(0.63,0.76) |
| Cycle 1 | |  |  | | 1.00 | | 1.00 |
| **Education*income** | |  | | |  | |  |
| 12 or < 12 years*low | | -0.24 [0.14] | -0.24 [0.14] | | 0.78(0.60,1.03) | | 0.78(0.60,1.03) |
| Overall 12 or <12 years*low income | | | | | 1.03 | |  |
| 12 or < 12 years*middle income | -0.07 [0.12] | | -0.07 [0.13] | | 0.93(0.74,1.17) | | 0.93(0.71,1.21) |
| Overall 12 or < 12 years*middle income | | | | | 1.21 | |  |
| **General Health*Sex** |  | | | |  | |  |
| Poor*female | 0.58 [0.21] | | 0.58 [0.22] | | 1.78(1.19,2.68) | | 1.78(1.15,2.76) |
| Overall Poor*female |  | | | | 43.91 | |  |
| Fair*female | 0.26 [0.15] | | 0.26 [0.16] | | 1.30(0.96,1.76) | | 1.30(0.95,1.77) |
| Overall Fair*female |  | | | | 11.85 | |  |
| Good*female | 0.16 [0.12] | | 0.16 [0.12] | | 1.17(0.92,1.50) | | 1.17(0.92,1.50) |
| Overall Good*female |  | | | | 4.96 | |  |
| Very good*female | 0.05 [0.12] | | 0.05 [0.11] | | 1.05(0.83,1.33) | | 1.05(0.84,1.31) |
| Overall Very Good*female |  | | | | 2.36 | |  |
| **General Health*household smoking** | | | | | | | |
| Poor*yes | -0.01 [0.21] | | -0.01 [0.23] | 0.98(0.65,1.49) | | 0.98(0.63,1.54) | |
| Overall Poor*yes |  | | | 22.22 | |  | |
| Fair*yes | 0.08 [0.14] | | 0.08 [0.16] | 1.09(0.82,1.44) | | 1.09(0.80,1.45) | |
| Overall Fair*yes |  | | | 7.30 | |  | |
| Good*yes | -0.13 [0.11] | | -0.13 [0.12] | 0.88(0.70,1.10) | | 0.88(0.70,1.10) | |
| Overall Good*yes |  | | | 2.75 | |  | |
| Very good*yes | -0.18 [0.11] | | -0.18 [0.11] | 0.83(0.67,1.04) | | 0.83(0.67,1.04) | |
| Overall Vary Good*yes |  | | | 1.71 | |  | |
